# A recombineering-based platform for high-throughput genomic editing in *Escherichia coli*

**DOI:** 10.1128/aem.00193-25

**Published:** 2025-06-12

**Authors:** Zeyu Liang, Chaoyong Huang, Yitian Li, Chao Yang, Ning Wang, Xiaoyan Ma, Yi-Xin Huo

**Affiliations:** 1 Center for Future Foods, Muyuan Laboratory, Zhengzhou, Henan Province, China; 2 Key Laboratory of Molecular Medicine and Biotherapy, Aerospace Center Hospital, School of Life Science, Beijing Institute of Technology 47833, Beijing, China; 3 Beijing Institute of Technology (Tangshan) Translational Research Center, Tangshan, Hebei, China; Centers for Disease Control and Prevention, Atlanta, Georgia, USA

**Keywords:** bacterial genomics, genomic editing, recombineering, high-throughput engineering

## Abstract

Functional analysis of bacterial genes or genomic fragments *in vivo* primarily relies on the analysis of knockout strains. Although various methods have successfully generated bacterial knockout mutants, the parallel operation of multiple sites, especially in biofoundries, remains challenging. New technological refinements of existing methods are necessary for high-throughput genomic editing in bacteria. In this study, to modify numerous sites in parallel, we optimized the linear donor DNA by adding modification at the different positions and achieved high-efficiency recombination with chemical transformation. Then, by combining with the CRISPR system, we established a guide sequence-independent and donor DNA-mediated genomic editing (GIDGE) method, enabling efficient and scarless engineering of common *E. coli* strains as well as wild-type strains such as *E. coli* MG1655, with particularly marked advantages demonstrated in *E. coli* Nissle 1917. This method allows for high-throughput genomic engineering in a 96-well format and is useful for sequence deletion with a wide range of lengths, sequence insertion, sequence replacement, and point mutation. As a proof-of-concept study, we constructed 96 single-gene knockout mutants and a genomic large-fragment deletion library in *E. coli* K-12 MG1655 using the GIDGE method. This high-throughput and easy-to-use method has great potential for automation and can be adapted for use in biofoundries.

**IMPORTANCE:**

With the increasing demand in the microbiology field and the expansion of its application scope, the urgency for genome editing techniques that are not only efficient and versatile but also capable of high-throughput processing and even automation has become increasingly critical. In this study, we enhanced the efficiency of recombination engineering by incorporating modifications and integrated it with the CRISPR system to develop an advanced gene editing method. This method allows for various gene editing events such as insertion, replacement, and long fragment knockout without the need for plasmid construction. It not only demonstrated high efficiency in common *E. coli* strains but also exhibited marked advantages in the probiotic strain *E. coli* Nissle 1917. This method is a versatile, efficient approach capable of high-throughput parallel gene editing. Using this method, we successfully constructed a large-scale strain library, significantly accelerating the process of microbial engineering.

## INTRODUCTION

The availability of complete bacterial genome sequences has provided a wealth of information on the molecular structure and organization of many genes and open reading frames (ORFs) whose functions are poorly understood. A systematic mutational analysis of genes and genomic fragments in their original locations could provide significant insights into their functions. To address the identification of key genes corresponding to genotype-phenotype relationships, parallel knockouts of multiple sites are frequently employed in genomic and metabolic engineering (1–4). An easy-to-use high-throughput genome engineering tool, aiming to circumvent labor-intensive plasmid construction processes, still needs to be developed.

In the early years, bacterial gene knockout was predominantly accomplished using non-replicating suicide plasmids (5). This method requires the construction of a specific suicide plasmid for each genomic editing and relies on a two-step homologous recombination process that involves a counterselection system. Counter selection by the *sacB* gene in the second recombination leads to a high false-positive rate (6) although the first recombination could be efficiently identified by antibiotic selection. The recombineering technique was proposed with the discovery of bacteriophage homologous recombination systems (7). Typically, Datsenko and Wanner exploited the high-efficiency lambda Red system and proposed a simple method for the one-step inactivation of chromosomal genes in *E. coli K-12* using PCR products (8). The basic strategy was to replace a chromosomal sequence with an antibiotic-resistant gene generated through PCR using primers with short homology extensions. This was accomplished by Red-mediated recombination in these flanking homologies. This method avoids the construction of specific plasmids and replaces them with PCR products. However, there are also several drawbacks. First, most bacteria are not readily transformable with linear DNA because of intracellular exonucleases that degrade linear DNA (9). Second, eliminating the antibiotic-resistant gene requires a helper plasmid expressing the FLP recombinase, which acts on the directly repeated FLP recognition target (FRT) sites flanking the resistance gene (10). Third, after eliminating the antibiotic-resistant gene, an FRT-contained sequence will be left as a scar. To facilitate the elimination of the antibiotic-resistant gene and achieve scarless editing, the I-SceI endonuclease was introduced (11–13). After replacing the target genomic sequence with a marker-containing linear DNA cassette, the I-SceI endonuclease was expressed to stimulate the deletion of the introduced marker by the double-strand break (DSB)-mediated intramolecular recombination. However, introducing the I-SceI endonuclease would decrease the efficiency of the first recombination due to the inevitable leaking expression of this enzyme.

Since the CRISPR technology was reported to manipulate bacterial genomes (14–17), the CRISPR technology, which relies on guide RNA (gRNA)-directed Cas protein cleavage at a specific protospacer sequence where a protospacer-adjacent motif (PAM) exists to kill wild-type cells, has been widely utilized (14, 18, 19). This technology avoids the reliance on selectable markers, site-specific recombination systems, and counter-selection systems. Although CRISPR-based genome editing techniques have been widely used in bacteria, there are still several drawbacks that need to be addressed to enhance its application. First, in point mutation experiments, additional mutations must be introduced into the protospacer or PAM to avoid post-editing DNA cleavage by CRISPR/Cas. Second, the commonly used systems often require extensive and labor-intensive plasmid construction processes.

To achieve efficient genome engineering, straightforward and versatile gene editing is fundamental. CRISPR technology is well-established, but recombination systems remain a bottleneck in strain engineering. In this study, we aim to enhance recombination efficiency and develop an easy-to-use tool for high-throughput *Escherichia coli* engineering that is site-independent and eliminates plasmid construction. To achieve this, we designed various donor DNA formats with varying strandedness (single/double-strand), end structure (blunt/sticky end), and single-strand formation (positive/negative ssDNA), based on existing recombination models (20–23). By optimizing the donor DNA, we increased the Red recombination efficiency by two to three orders of magnitude, allowing for chemical transformation to generate enough recombinants. Since the throughput of current electroporation experiments is limited, chemical transformation, which only requires temperature control, is more convenient and adaptable to a 96-well plate format, thereby improving the throughput of precise genome editing. Subsequently, the CRISPR/Cas9 system was used to eliminate selectable markers. The CRISPR/Cas9 system works through the cooperation of Cas9 protein and single-guide RNA (sgRNA), and when both are controlled by inducible promoters, it significantly reduces unintended DNA cleavage. The method we established was called GIDGE (Guide sequence-Independent and donor DNA mediated Genomic Editing), which is efficient for scarless genomic engineering, including sequence deletion with a wide range of lengths, sequence insertion, sequence replacement, and point mutation. Based on the GIDGE method, genomic editing was carried out in 96-well plates throughout the entire process, which demonstrated that the GIDGE method could quickly obtain massive knockout mutants, including single-gene knockout mutants and large-fragment deletion mutants.

## RESULTS

### Evaluation of recombination efficiency using different DNA donors

Homologous recombination is a crucial mechanism that participates in DNA repairing and maintains genome integrity, which could be combined with the CRISPR system to modify the organisms. Previously, there was a scientific consensus in the recombineering community that Lambda Red recombination initiates with the degradation of the 5′ ends of dsDNA by the exonuclease (Exo) of the Lambda Red system. The single-strand binding protein (SSB) protects the ssDNA from digestion by Exo, thereby facilitating recombination (24, 25). In addition, ssDNA could also achieve recombination with the aid of the recombination system (26). However, the relative efficiencies of different DNA forms under uniform conditions remain unclear. To address this gap, we designed a series of DNA donors varying in strandedness (single/double-strand), end structure (blunt/sticky end), and single strand formation (positive/negative ssDNA). By comparing their recombination efficiencies, we aimed to identify the optimal donor design for enhancing CRISPR-based editing.

**Fig 1:**
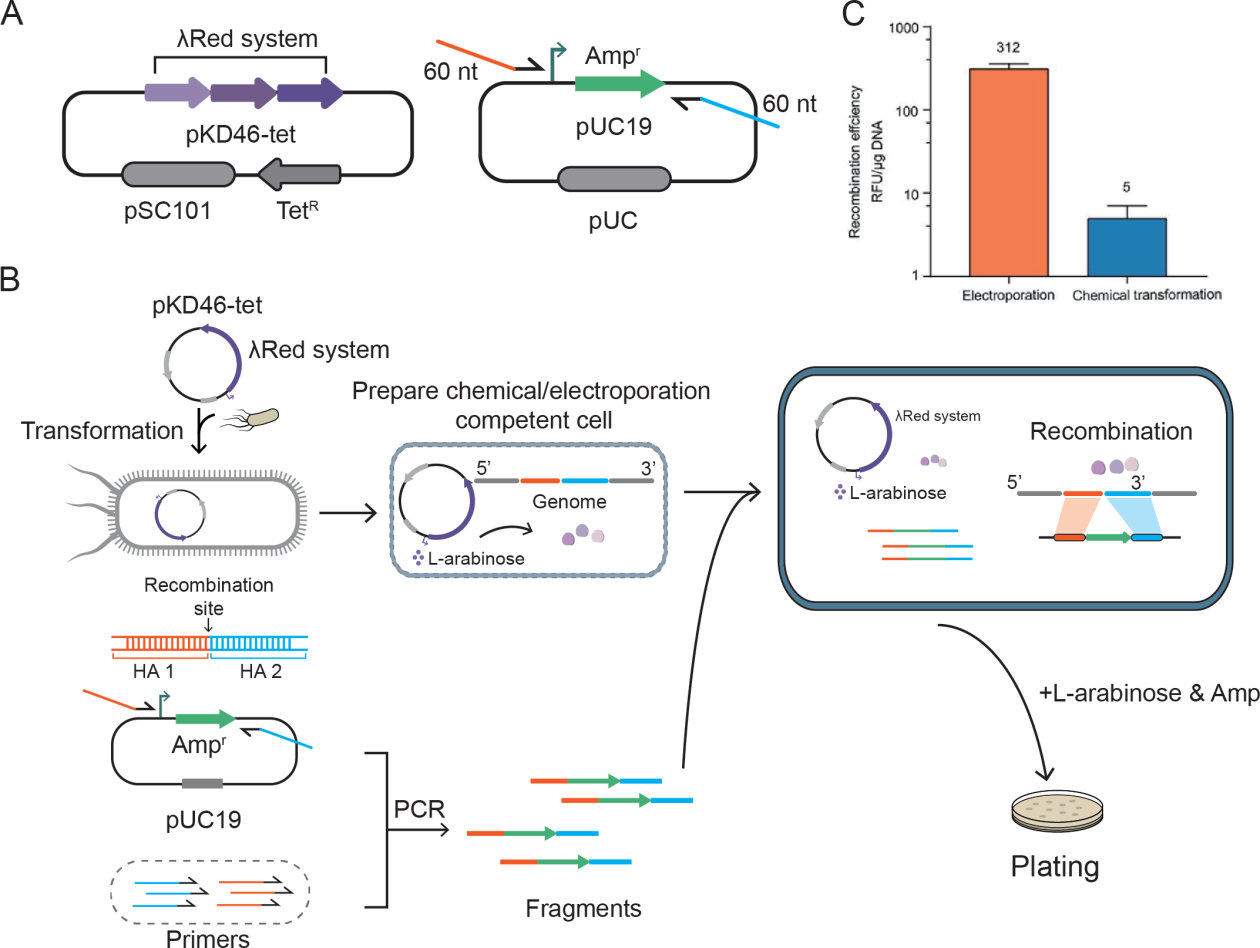
Red recombination tests using unmodified donor DNA. (**A**) Plasmids used for recombination tests. The *pKD46-tet* was used for expressing Red recombinases. The *pUC19* was used as the PCR template to amplify donor DNA. (**B**) Illustration of the procedures for Red recombination tests. (**C**) Results of Red recombination tests using electroporation and chemical transformation. Data are expressed as means ± S.D. from over three independent experiments.

We designed a recombineering test using two plasmids named *pKD46-tet* and *pUC19* (Fig. 1A). The plasmid *pKD46-tet* is a variant of *pKD46*, containing the lambda Red recombinase system. The *pUC19* is a vector, containing an Amp-resistant (Amp^r^) gene, which provides the selection marker for recombination. With these two plasmids, we carried out the following recombineering test. The recombination efficiency was tested on the *E. coli* JM109 strain, and we chose the *lacZ* as the target gene to evaluate the recombination efficiency by recombinant forming units per microgram of donor DNA (RFU/μg DNA). The colonies could grow on the LB plate containing ampicillin, which means the achievement of recombination. Thus, recombination efficiencies equal the colony number formed per microgram of donor DNA times positivity rate. An unmodified double-stranded DNA (dsDNA), referred to as type-I donor, was amplified from *pUC19* to target the *lacZ* gene. This dsDNA contains an ampicillin resistance (Amp^r^) gene flanked by two homologous arms (HAs), with no additional functional elements. The sequences of these homologous arms were identical to those upstream and downstream of the recombination site of *lacZ* gene, which were used for homologous recombination of the fragments (Fig. 1B).

First, we investigate the difference between electroporation and chemical transformation method on recombination efficiency when using the type-I donor. For each recombineering test, 20 colonies were randomly picked for PCR verification. The recombinants generated a 1,349 bp amplicon, and the unedited *JM109* generated a 570 bp amplicon (Fig. 1B). The PCR results showed that all tested colonies got the 1,349 bp amplicon (Fig. S1), indicating that the positivity rates were 100%. The electroporation produced a high editing efficiency of 312 RFU/μg DNA, and the chemical transformation had a low editing efficiency of 5 RFU/μg DNA (Fig. 1C).

**Fig 2:**
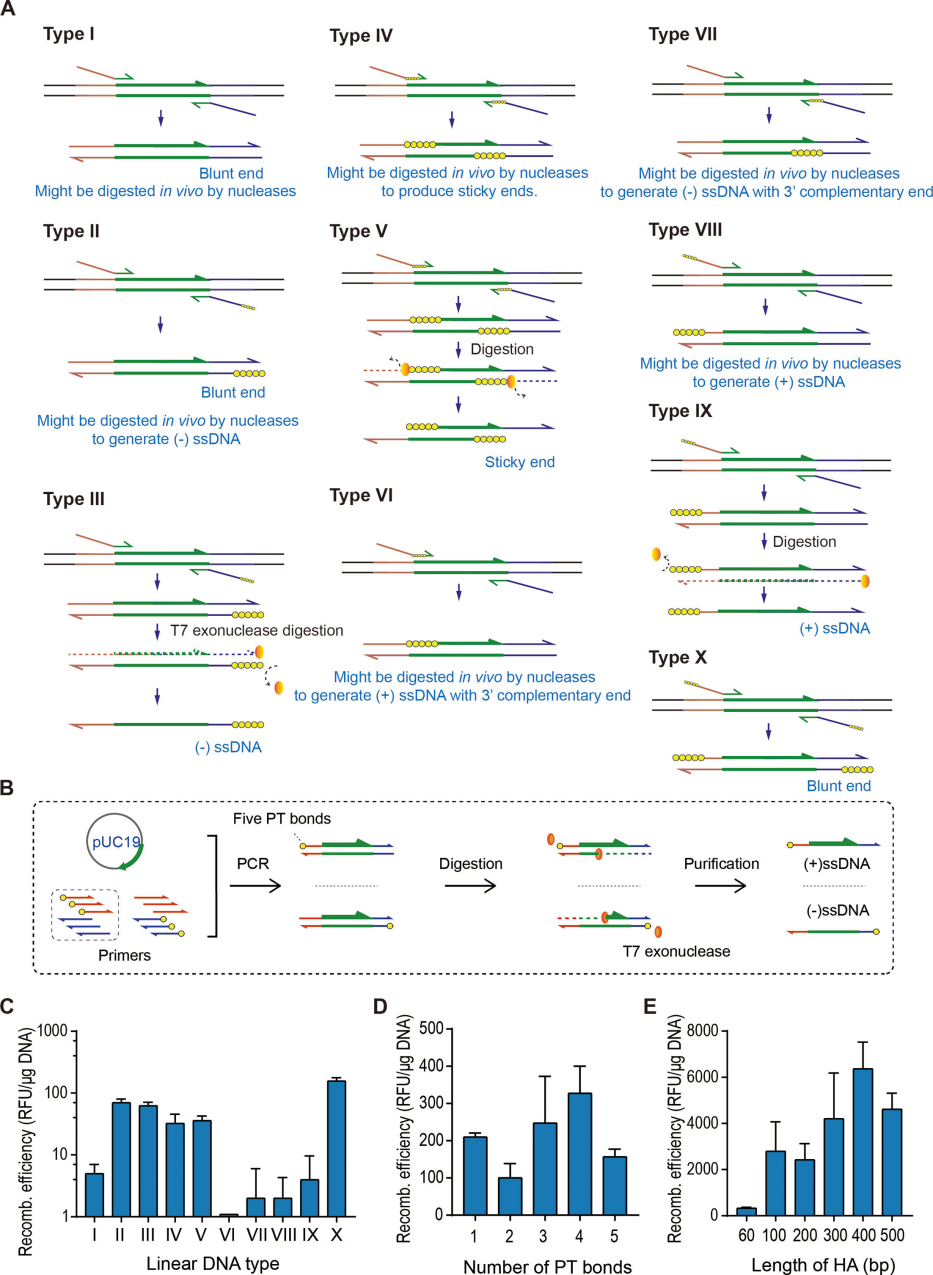
Optimization of donor DNA. (**A**) Different types of donor DNA used for Red recombination tests. The type II to type X contains PT modifications on donor DNA. (**B**) Procedures of preparing single-stranded donor DNA. (**C**) Results of Red recombination tests using different types of donor DNA. (**D**) Results of Red recombination tests using donor DNA with varying numbers of PT bonds. (**E**) Results of Red recombination tests using donor DNA with HAs of different lengths.

To investigate how DNA donor architectures influence Red recombination, we engineered ten distinct repair templates with controlled structural variations (Fig. 2A). These donors were designed by introducing phosphorothioate (PT) modifications—a chemical alteration replacing non-bridging phosphate oxygens with sulfur in the DNA backbone. This substitution confers resistance to exonucleases and endonucleases that target natural phosphate bonds, thereby protecting linear DNA from degradation (27, 28). The enhanced metabolic stability of PT-modified DNA has been leveraged to achieve efficient-targeted integration in mammalian (29) and plant cells (30), demonstrating its broad applicability in genome engineering. T7 exonuclease, a dsDNA-dependent 5′ exonuclease, was used to digest the template dsDNA for generating these ten different DNA donors *in vitro*. After digestion and purification, the designed ten types of donor DNA could be obtained (Fig. 2B). Such as the type-IV donor, five PT bonds were placed consecutively on both strands of the dsDNA at positions downstream of the HAs, and the type-V donor, a partial dsDNA with 3′ overhangs, was prepared by digesting the type-IV donor *in vitro* with T7 exonuclease. Other dsDNA could be obtained using PT-modified primers amplified from template plasmid.

The ten different types of donor DNA were individually transformed into the *JM109/pKD46-tet* by chemical transformation. As a result, the type-II donor, type-III donor, type-IV donor, type-V donor, and type-X donor generated much higher recombination efficiencies than the type-I donor, and these editing efficiencies were 70 RFU/μg DNA, 62 RFU/μg DNA, 33 RFU/μg DNA, 36 RFU/μg DNA, and 157 RFU/μg DNA, respectively. (Fig. 2C). Overall, in the presence of PT modification, the Type-X donor exhibited the highest recombination efficiency, which was 19.6 times higher than the Type-I donor.

To improve the recombination efficiencies, we further optimized the type X donor. First, we optimized the number of PT bonds. A series of dsDNA fragments with 60 bp HAs containing one to five consecutive PT bonds on both 5′ ends were used for recombination. The results showed that the dsDNA with four consecutive PT bonds generated the highest editing efficiency, 328 RFU/μg DNA (Fig. 2D), higher than the one (312 RFU/μg DNA) produced by electroporation using the type I donor. The editing efficiency produced by chemical transformation could be further improved by optimizing the length of HAs. A series of dsDNA fragments with four consecutive PT bonds on both 5′ ends and 60 bp, 100 bp, 200 bp, 300 bp, 400 bp, and 500 bp HAs were used for recombination. The results showed that the dsDNA fragments with 100 bp, 200 bp, 300 bp, 400 bp, and 500 bp HAs generated much higher editing efficiencies than the dsDNA fragment with 60 bp HAs. The dsDNA with 400 bp HAs generated the highest editing efficiency, 6,370 RFU/μg DNA (Fig. 2E). Under the condition of the same 400 bp homologous arms, we compared the recombination efficiency between modified and unmodified donors. The results showed that the recombination efficiency of the modified Donor was 1.47 times higher than that of the unmodified Donor (Fig. S2).

### Establishment of the GIDGE method

**Fig 3:**
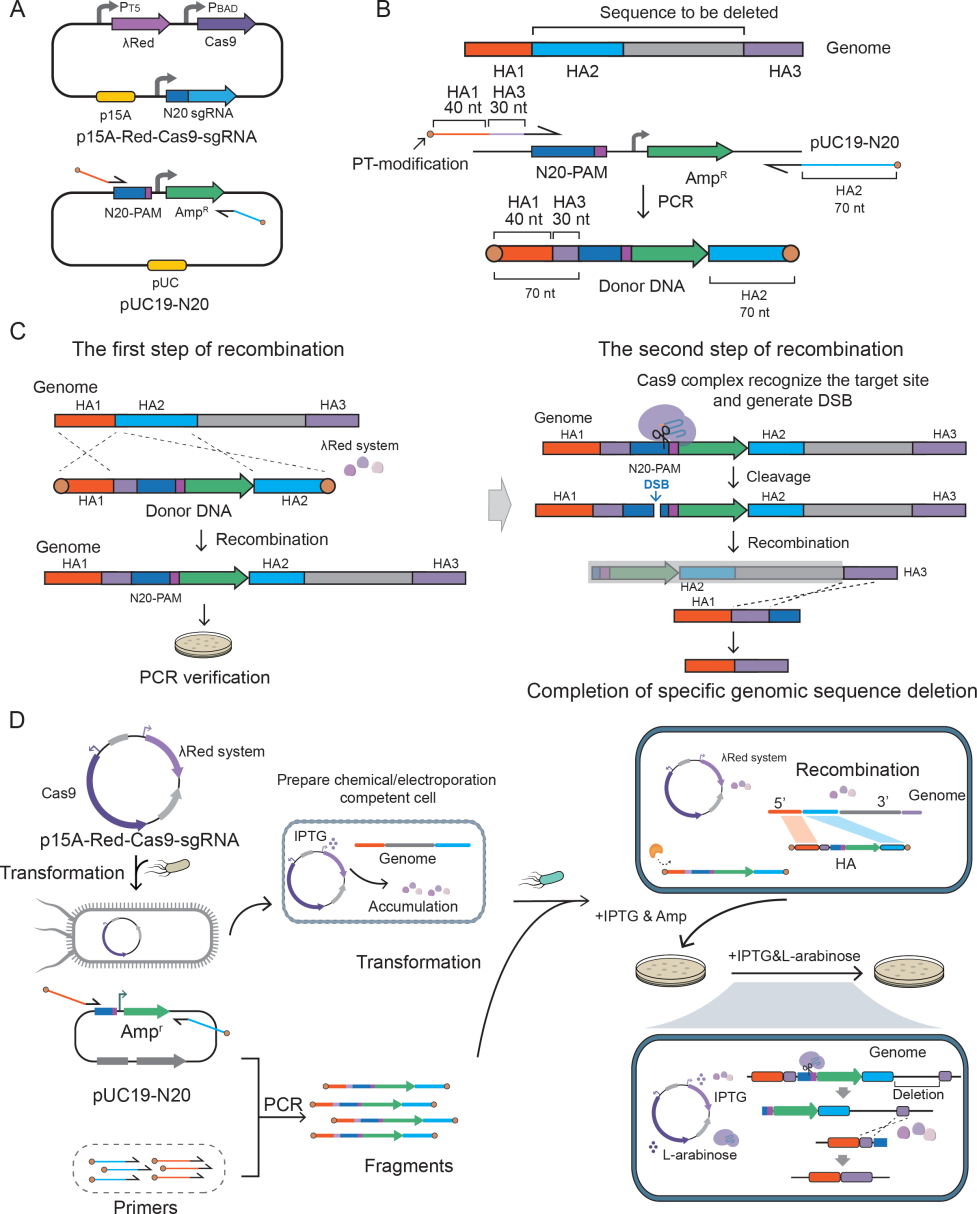
Genomic editing with the GIDGE method. (**A**) Plasmids used in the GIDGE method. (**B**) Preparation of donor DNA with HAs for sequence deletion. When preparing donor DNA with short HAs, a pair of 90-nt primers were designed. Each primer contained a 20-nt prime sequence and a 70-nt overhang. The upstream primer was designed to include sequences homologous to HA1 and HA3, while the downstream primer contained sequences homologous to HA2. (C) The process of achieving the deletion of specific sequences with the help of two-step homologous recombination. Using *pUC19-N20* as a template, PCR-amplified donor DNA was generated for genomic integration. After the first step of recombination, the Cas9 complex could recognize the target site, inducing a DSB that triggers subsequent recombination. After the second recombination step, the specific sequence could be deleted from the genome. (**D**) Schematic diagram of GIDGE method for sequence deletion.

Based on the optimized recombination system, we exploited to establish the GIDGE method for genomic engineering. The vector *p15A-Red-Cas9-sgRNA* containing a medium-copy-number *p15A* replicon was used to express the CRISPR and recombination systems. The lambda Red system was expressed under the control of a T5 promoter, which is inducible by IPTG, while Cas9 protein and sgRNA were driven by a P_BAD_ promoter activated by L-arabinose. Additionally, the plasmid included an sgRNA sequence designed to recognize the pre-designed target site. The *pUC19-N20* contained a high-copy-number *pUC* replicon, an Amp^r^ gene, and a pre-designed target site containing 20 bp artificial CRISPR target (referred to as N20) with its PAM sequence, which was non-homologous to the *E. coli* genome (Fig. 3A).

To demonstrate donor DNA preparation, we used sequence deletion as an example. When using short HAs, we designed 90-nt primers containing 20-nt priming sequences and 70-nt overhangs: the upstream primer was homologous to HA1 and HA3, and the downstream primer was homologous to HA2 (Fig. 3B). Using *pUC19-N20* as template, PCR- amplified donor DNA was generated for genomic integration. Following the first step recombination, the Cas9 complex recognized the pre-designed target site, inducing a DSB that triggers the subsequent second step of recombination (Fig. 3C). The GIDGE method accommodates both short (Fig. 3B) and long HA (Fig. S3) donor DNAs, enabling diverse genomic edits including deletions, insertions, replacements, and point mutations. The editing process involves transforming donor DNA into cells containing *p15A-Red-Cas9-sgRNA*, where initial recombination integrates the donor DNA, followed by Cas9-mediated DSB formation that activates secondary recombination (Fig. 3D). For long HAs, we designed primers to generate functional fragments, which for insertions or replacements were assembled via SOE-PCR (Fig. S4). Complete protocols for all editing types were detailed in Fig. S5.

### Large-fragment deletion in *E. coli* MG1655 and *E. coli* Nissle 1917

**Fig 4:**
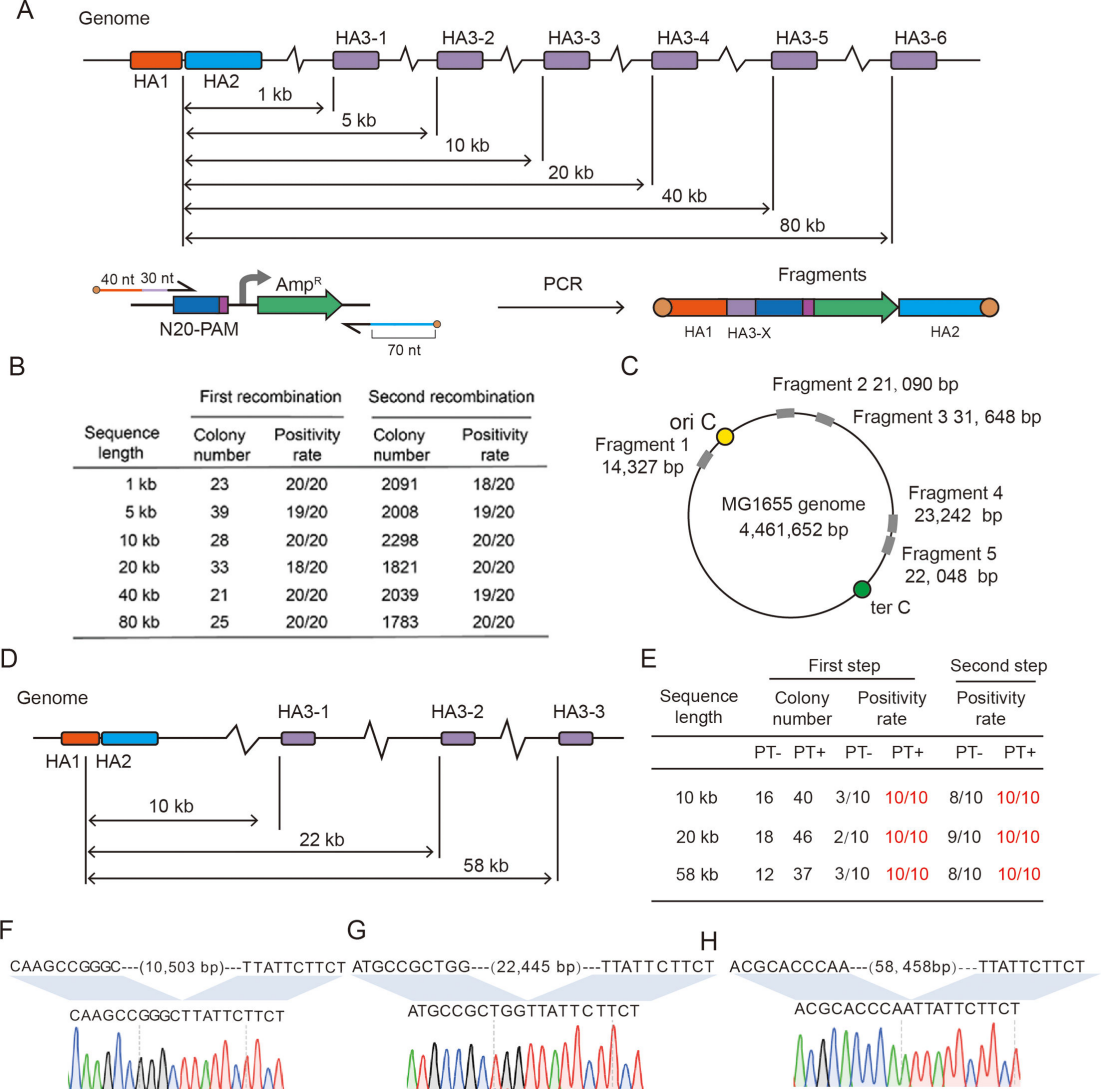
Large-fragment deletion in *E. coli* MG1655 and *E. coli* Nissle 1917. (**A**) Genomic editing tests of sequence deletion of different lengths in MG1655. (**B**) *E. coli* MG1655 genomic editing results of sequence deletion of different sizes. (**C**) Deletion of *E. coli* MG1655 large fragments in different genomic locations. (**D**) Genomic editing tests of sequence deletion of different lengths in EcN. (**E**) EcN genomic editing results of sequence deletion of different sizes. (F through H) The sequencing results of 10 kb, 22 kb, and 58 kb fragment deletions.

To show the ability of the GIDGE method in sequence deletion of different lengths, we selected six sequences in the *MG1655* genome and deleted them individually. MG1655 approximates wild-type *E. coli* (31) which has a low competency. The lengths of these sequences were 1 kb, 5 kb, 10 kb, 20 kb, 40 kb, and 80 kb, respectively (Fig. 4A). These sequences start at the same genomic site, 286,778. By consulting the EcoCyc, a model-organism database for MG1655 (32), we knew these sequences had no growth-essential gene. Donor DNA fragments with short HAs were used for these sequence deletions. These donor DNA fragments were similar, and the only difference was that their HAs were homologous to different regions of the MG1655 genome. The colony numbers and positivity rates of the first recombination and the second recombination in these sequence deletions were shown in Fig. 4B. The results showed that the length of deleted sequence has no significant influence on the editing efficiency, indicating that the GIDGE method was an efficient tool for large-fragment deletion. Then, we selected five large fragments in different locations of the MG1655 genome and deleted them individually (Fig. 4C). The lengths of these fragments were 14.3 kb, 21.1 kb, 31.6 kb, 23.2 kb, and 22.0 kb, respectively. By consulting the EcoCyc database, we determined that these fragments contained no growth-essential gene. As expected, these fragments were deleted successfully and efficiently (Table 1).

**TABLE 1:** Genomic editing carried out in this study^*a*^

Strain	Editing type	Content	Trans. Effi.	Colony no.	Positivity rate
JM109	Sequence deletion	Δ*lacZ*	5.4 × 10^6^	287	20/20
JM109	Sequence deletion	Δ*xthA*	5.4 × 10^6^	226	18/20
DH10B	Sequence deletion	Δ*recBCD*	6.5 × 10^6^	234	20/20
DH10B	Sequence replacement	P_T5_-*recET*	6.5 × 10^6^	325	20/20
JW128	Sequence deletion	Δ*tnaA*	2.9 × 10^6^	308	20/20
JW128	Point mutation	*trpE* ^fbr^	2.9 × 10^6^	243	17/20
JW128	Sequence insertion	P_tac_-*trpE*	2.9 × 10^6^	192	19/20
JW128	Sequence deletion	Δ*frdBC*	2.9 × 10^6^	296	20/20
JW128	Sequence deletion	Δ*pflB*	2.9 × 10^6^	304	20/20
JW128	Sequence deletion	Δ*fnr*	2.9 × 10^6^	323	19/20
JW128	Sequence deletion	Δ*adhE*	2.9 × 10^6^	376	20/20
JW128	Sequence deletion	Δ*pta*	2.9 × 10^6^	268	17/20
JW128	Sequence deletion	Δ*ldhA*	2.9 × 10^6^	411	20/20
JW128	Sequence deletion	Δ*ldhA*	2.9 × 10^6^	264	20/20
MG1655	Sequence deletion	Δ*recA*	3.7 × 10^5^	32	20/20
MG1655	Sequence deletion	Δ*endA*	3.7 × 10^5^	43	20/20
MG1655	Sequence deletion	Δ*mcrA*	3.7 × 10^5^	29	20/20
MG1655	Sequence deletion	Δ(*mcrCB-hsdSMR-mrr*)	3.7 × 10^5^	36	16/20
MG1655	Sequence replacement	Δ*araBAD*::Tet^r^	3.7 × 10^5^	22	20/20
MG1655	Sequence deletion	Δ*umuDC*	3.7 × 10^5^	27	15/20
MG1655	Sequence deletion	Δ1 kb	3.7 × 10^5^	23	18/20
MG1655	Sequence deletion	Δ5 kb	3.7 × 10^5^	39	19/20
MG1655	Sequence deletion	Δ10 kb	3.7 × 10^5^	28	20/20
MG1655	Sequence deletion	Δ20 kb	3.7 × 10^5^	33	20/20
MG1655	Sequence deletion	Δ40 kb	3.7 × 10^5^	21	19/20
MG1655	Sequence deletion	Δ80 kb	3.7 × 10^5^	25	20/20
MG1655	Sequence deletion	Δ14.3 kb	3.7 × 10^5^	33	20/20
MG1655	Sequence deletion	Δ21.1 kb	3.7 × 10^5^	41	20/20
MG1655	Sequence deletion	Δ31.6 kb	3.7 × 10^5^	28	18/20
MG1655	Sequence deletion	Δ23.2 kb	3.7 × 10^5^	39	20/20
MG1655	Sequence deletion	Δ22.0 kb	3.7 × 10^5^	21	19/20
EcN	Sequence deletion	Δ10.5 kb	4.2 × 10^5^	40	10/10
EcN	Sequence deletion	Δ22.4 kb	4.2 × 10^5^	46	10/10
EcN	Sequence deletion	Δ58.45 kb	4.2 × 10^5^	37	10/10

^*a*^ Transformation efficiencies (CFU/μg DNA) were tested with the pUC19 plasmid. Colony number and positive rate are the results of the first-step recombination.Trans. Effi., transformation efficiency; Colony No., colony number.

Common *E. coli* strains JM109, DH10B, JW128 (33), and MG1655 were used to test the GIDGE method. All genomic editing tests in this study were listed in Table 1. In these tests, donor DNA fragments with short HAs were used. In the GIDGE method, the editing efficiency depends on the first recombination. Therefore, the colony number and positivity rate generated by the first recombination were listed (Table 1). In these tests, transformation efficiencies of more than 10^5^ CFU/µg *pUC19* were sufficient. If necessary, donor DNA with long HAs could be used to improve the editing efficiency further. The GIDGE method was convenient and time-saving. On the one hand, this method was site-independent and released the burden of constructing plasmids for each editing. On the other hand, this method was electroporation-independent and scarless. Only 3*n* + 2 days were needed for multi-round genomic editing (Fig. S6).

The *E. coli* Nissle 1917 (EcN) is a gram-negative probiotic, and this strain has recently seen its application scope broadened through its engineering into living drugs, industrial platforms, and drug delivery systems (34–36). As a subtype of *E. coli*, EcN is capable of undergoing genetic engineering utilizing the same tools previously employed in *E. coli* K-12. To test the GIDGE method in, we analyzed the genome of EcN and selected three large fragments for deletion. The lengths of these sequences were 10 kb, 20 kb, and 58 kb, respectively (Fig. 4D). We compared the editing efficiencies of the unmodified and PT-modified fragments within EcN. The results demonstrated that PT modifications not only increased the RFU compared to the unmodified fragments but also significantly enhanced the editing efficiencies (Fig. 4E) and that the GIDGE method could be used to delete large fragments in EcN (Fig. 4F through H). The growth condition of these strains was tested, and the results were showed in Fig. S7.

These results indicated that the GIDGE method was a straightforward editing approach for MG1655 strain without the need for plasmid construction. For EcN strain, incorporating PT modification at the ends of homologous arms significantly boosted editing efficiency, enabling highly effective large fragment deletions and enhancing overall gene editing efficiency.

### Parallel high-throughput gene editing in a 96-well format

**Fig 5:**
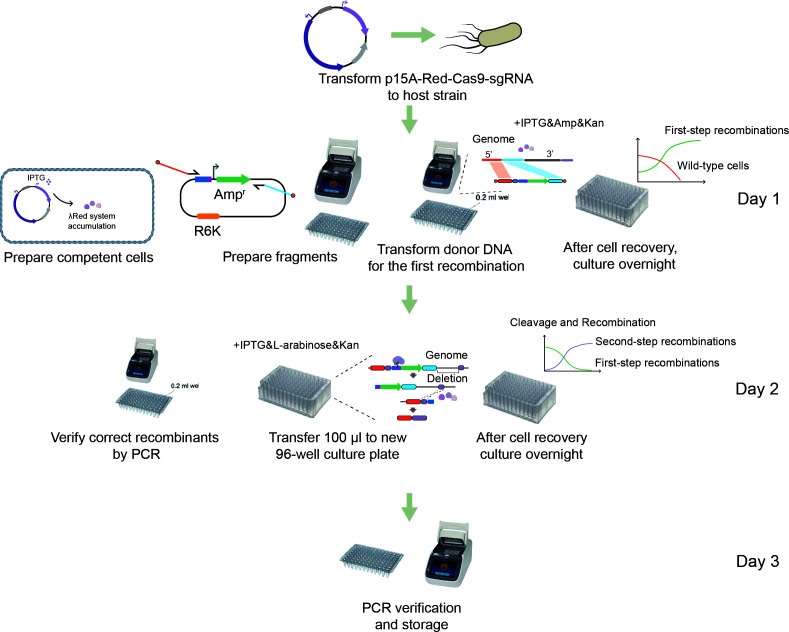
Procedures of high-throughput genomic editing in parallel based on the GIDGE method.

The GIDGE method enabled us to obtain high editing efficiency in multiple strains, and it was adapted into a 96-well format to achieve parallel operation of multiple sites for high-throughput engineering (Fig. 5). All operations were performed in 96-well PCR plates and 96-well culture plates. Here, we take the deletion of the JM109 *lacZ* gene as an example to test the editing process. First, 2.5 µL of donor DNA was added into each well of a 96-well PCR plate containing 25 µL of *JM109/p15A-Red-Cas9-sgRNA* chemically competent cells in each well. To simplify the preparation of donor DNA, we omitted the DNA purification operations and directly added PCR products. To avoid the influence of PCR buffer on competent cells, the volume of the PCR products should not exceed 1/10 of the total volume. A problem arose because the template plasmid *pUC19-N20* remaining in the PCR products would lead to many wild-type cells. To solve this problem, we replaced the *pUC* replicon of the *pUC19-N20* with the *R6K* replicon, generating *pR6K19-N20*. Replication of plasmids harboring the *R6K* replicon is dependent on the pir gene-encoded π protein (37). Therefore, the *pR6K19-N20* could replicate only in engineered strains carrying the pir gene, such as DH5α λpir. We could use DH5α to construct and harbor the *pR6K19-N20* plasmid. As the other *E. coli* strains to be edited in our experiments were pir-free, the template plasmid *pR6K19-N20* could not survive in them, thus avoiding the growth of wild-type cells. After mixing well, the transformation experiments were conducted in 96-well PCR plate, and after heat shock, 100 µL of SOC medium preheated at 37℃ was added into each well. The cells could recover in the 96-well culture plate in a 37℃ shaker for 2 hours. During this period, the first recombination occurred, and the Amp^r^ cassette was inserted into the genome. Then, Ampicillin and Kanamycin were added into each well, and the 96-well culture plate was incubated overnight in a 37℃ shaker to kill the wild-type cells and enrich the first-step recombinants. The next day, the culture was inoculated to a new 96-well culture plate, and 900 µL of LB medium and Kanamycin was added into each well. The 96-well culture plate was incubated in a 37℃ shaker to activate cells. After cultivation, IPTG and L-arabinose were added to each well, and the 96-well culture plate was incubated in a 37℃ shaker. During this period, the second recombination occurred, and the Amp^r^ cassette and the *lacZ* gene were deleted from the genome. After that, 50 µL of culture was transferred to a new 96-well culture plate, and 950 µL of LB medium and L-arabinose were added into each well and incubated overnight in a 37℃ shaker to kill the escaped cells and enrich the second-step recombinants. By this point, the genomic editing had been completed. To detect the results, 1 µL of culture was taken from each well for PCR verification. The results showed that all 96 parallel gene deletion tests were successful (Fig. S8), which indicated the good repeatability of the GIDGE method in 96-well format. To preserve the edited strains, 500 µL of 50% glycerol was added into each well, and the 96-well culture plate was stored below −70℃. When a plasmid-free edited strain was needed, a few preserved cultures were taken for streaking at an LB plate containing sucrose. Colonies growing on the plate were plasmid-free.

### Construction of 96 single-gene knockout mutants and a large-fragment deletion library

**Fig 6:**
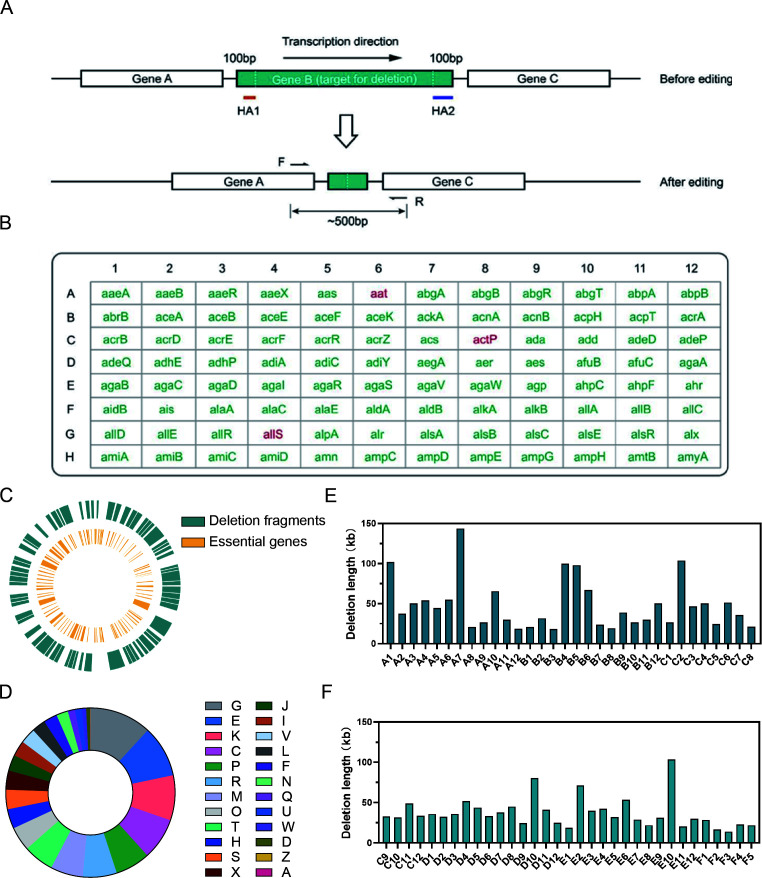
Construction of 96 single-gene knockout mutants and a large-fragment deletion library by the SHE platform. (**A**) Strategy of in-frame gene knockout. (**B**) Arrangement of gene deletions in the 96-well plate. (**C**) The deleted large fragments in MG1655. The inner yellow lines indicate the position of essential genes. The deleted large fragments were indicated in blue. (**D**) Characterization of COG categories for all deleted genes in the large-fragment deletion library. A: RNA processing and modification; C: Energy production and conversion; D: Cell cycle control and mitosis; E: Amino acid metabolism and transport; F: Nucleotide metabolism and transport; G: Carbohydrate metabolism and transport; H: Coenzyme metabolism; I: Lipid metabolism; J: Translation; K: Transcription; L: Replication and repair; M: Cell wall/membrane/envelop biogenesis; N: Cell motility; O: Post-translational modification, protein turnover, chaperone functions; P: Inorganic ion transport and metabolism; Q: Secondary structure; R: General functional prediction; S: Function Unknown; T: Signal transduction; U: Intracellular trafficking and secretion; V: Defense mechanisms; W: Extracellular structures; X: Mobilome: prophages, transposons Z: Cytoskeleton. (E and F) The size of the 65 deleted large-fragments constructed in this study.

To test the ability of the GIDGE method in high-throughput genomic editing, we selected 96 genes in the *MG1655* genome to make single-gene knockout mutants simultaneously. These genes are included in the Keio collection (1), so they are all growth-nonessential in LB medium. All deletions were within the ORFs, and the 100 bp sequences of the beginning and ending of the ORFs were reserved (Fig. 6A), which could avoid potential impacts on adjacent genes. After genomic editing, the cultures in 96 wells were tested by PCR to verify knockout mutations. For each gene knockout, a pair of primers were designed to target the upstream and downstream regions of the ORF (Fig. 6A). An amplicon of around 500 bp could be obtained if the target gene was deleted. In our first attempt, 93 knockout mutants (marked in green) were obtained (Fig. 6B). The deletion of genes *aat*, *actP*, and *allS* (marked in red) was not completely fail. In these three deletions, amplicons of edited cells and wild-type cells were both obtained (Fig. S9), indicating that correct recombinants and unwanted cells coexisted in the cultures. This phenomenon was probably due to off-target recombination of the donor DNA in some cells. For these three genes, knockout mutants were separated by plate streaking of the cultures. The growing colonies were screened by PCR to obtain correct recombinants.

Then the multiple gene operation platform was utilized to construct a large-fragment deletion library of the MG1655 strain. First, 410 potentially essential genes were labeled in the *MG1655* genome map (Fig. 6C). We found that there were many long sequences harboring no potentially essential gene in the genome. These sequences were candidate nonessential fragments. Initially, we selected 81 large fragments. In our first attempt, 65 knockout mutants were obtained (Fig. 6C). No correct amplicon was obtained for the failed 18 fragments, implying that these fragments were growth essential. To further confirm whether these fragments were essential or not, we used the GIDGE method to delete them individually. We found that the first recombination was successful, but the second one failed. Since the second recombination is very efficient, a knockout mutant could be easily obtained if the target sequence is nonessential. Therefore, these 18 fragments were essential for growth.

To achieve a more comprehensive insight into the large fragment knockout library, we analyzed the deleted chromosome fragments. The deleted genomic regions of this library contain 2,627 *E. coli* MG1655 genes which could be grouped into 24 COG categories, among which carbohydrate metabolism and transport (G, 11.83%), amino acid metabolism and transport (E, 9.85%), transcription (K, 8.60%), energy production and conversion (C, 7.99%), and inorganic ion transport and metabolism (P, 6.66%) rank the top five categories with annotated functions (Fig. 6D). The total length of the knocked-out genes in *E. coli* is about 2,769 kb, accounting for 59.67% of the genome. In this large-fragment deletion library, the length of the designed segments ranges from 14.3 kb to 143.9 kb (Fig. 6E and F). Based on the large-fragment deletion library, we have screened out some gain-of-function phenotypes, such as mutants producing higher titer of amino acids (38), mutants tolerant to higher alcohols (39), and mutants with low plasmid loss (40).

## DISCUSSION

To develop a robust high-throughput engineering protocol, we first needed to establish an operation-friendly genomic editing method. Here, an operation-friendly method should meet two conditions: first, there is no need to construct plasmids, and second, only one transformation is required. Generally, methods based on suicide plasmids (41, 42) or CRISPR/Cas technologies (15, 43) involve plasmid construction, which increases the cost and difficulties for the operators. The lambda Red recombineering-based methods require linear DNA that could be easily prepared by a single PCR operation (7). Synthesized single-stranded DNA (ssDNA) can be used as the repair homologous arms to achieve gene editing. However, this strategy is more suitable for short fragment deletions, and there is no reported successful case for large fragment deletion (44, 45). We also tried to use ssDNA for long fragment deletion, but it failed.

The CRISPR/Cas9 system works by the Cas9-sgRNA complex that is encoded by two genes, which could significantly decrease leaking cleavage of the target site when both genes are controlled by strictly inducible promoters. Here, the CRISPR/Cas9 system was used as a specific endonuclease rather than a programmable DNA cleavage tool. A specific CRISPR target site (N20-PAM) was added to the Amp^r^ cassette so that this target was integrated into the genome along with the selectable marker by Red recombination. Then, the Cas9-sgRNA complex was induced to recognize the target site and cleave within it to trigger the intramolecular recombination during which the selectable marker was eliminated. Currently, Red recombineering methods rely on transformation efficiency to obtain enough recombinants (46, 47). In this study, we achieved high-efficiency Red recombination with chemical transformation by optimizing the donor DNA. The editing efficiency was increased by 65.6 times when a dsDNA with four consecutive PT bonds on both 5′ ends was used for recombination. This efficiency was enough in most cases, and when needed, the editing efficiency could be further increased by 19.4 times using a donor DNA with longer HAs of around 400 bp. The cost of PT modification has now been reduced to a low level.

The GIDGE method we established is useful and efficient for different types of genomic editing, including sequence deletions, sequence insertions, sequence replacements, and point mutations, which has been shown by many applications in this study. Especially, the GIDGE method was efficient for large-fragment deletions, and the fragment length did not affect the efficiency. Previously, many CRISPR/Cas-based methods were reported to be able to delete large fragments, and these methods, using homologous recombination (17, 48) or non-homologous end joining (16, 49), required plasmid construction. When using the GIDGE method, no plasmid was needed to construct, and the only DNA material needed to prepare was a linear dsDNA that could be easily obtained by a single PCR reaction.

The GIDGE method not only enabled efficient and scarless engineering of common *E. coli* strains such as JM109, DH10B, as well as wild-type strains such as *E. coli* MG1655 and *E. coli* Nissle 1917 (EcN). The EcN is a gram-negative probiotic, and this strain has been widely used in various application fields. However, due to the cell being recognized and digested by the internal defense system of the cell, the gene editing process in EcN is often inefficient. By integrating the PT-modification, the GIDGE method is a “user-friendly” approach in common *E. coli* strains, eliminating the reliance on vectors and high transformation efficiency of cells. In EcN, a non-model *E. coli* strain, the GIDGE method remains a notably efficient gene editing strategy, allowing for the effective deletion of large fragments, achieving comparable efficiency to that of other *E. coli* strains. For the EcN, compared to common *E. coli* strains, the genomic studies are relatively limited, which, in turn, restricts the accuracy of the prediction results of software, affecting the editing efficiency of the CRISPR system in this strain. However, the GUDGE method, by introducing highly efficient target sites through the recombination with the combination of the PT modifications, eliminates this limitation and provides a universal approach for editing EcN.

By using the R6K replicon (37) for the template plasmid, we could avoid the false-positive clones produced by the template plasmid remaining in the PCR products. In the GIDGE method, the selectable marker could be quickly eliminated by inducing DNA cleavage and intramolecular recombination, and another transformation to introduce a helper plasmid (13) or a new linear DNA (50) is unnecessary. Only 3*n* + 2 days were needed for multiple rounds of genomic editing when using the GIDGE method. Notably, this time includes preparation of donor DNA, genomic editing, and elimination of selectable markers and plasmids. The GIDGE method was not limited by the selection of target sites, which is a common advantage for recombineering-based methods compared with CRISPR/Cas-mediated methods. Taken together, the GIDGE method is site-independent, operation-friendly, scarless, and efficient. The limitation of the GIDGE method is that it could not edit essential genes because inserting the selectable marker in the first recombination will inactivate the target gene.

The operation-friendly and efficient nature of the GIDGE method enabled the establishment of the parallel gene editing operation platform with which high-throughput genomic editing could be achieved. By utilizing automated equipment, the strain construction cycle would be improved, and furthermore, this advancement could lead to increased throughput and reduced manual intervention. As the proof of concept, in this study, we utilized the parallel gene editing operation platform to construct 96 single-gene knockout mutants and a large-fragment deletion library to show its ability in high-throughput genomic editing. The large-fragment deletion library has many uses. For example, our team has used this library to screen for wanted phenotypes and to study the simplification of the *E. coli* genome (38–40). Multiple 96-well plates could be used to carry out more genomic editing simultaneously. Baba et al. reported the construction of 3985 *E. coli K-12* single-gene knockout mutants, the Keio collection (1), by using the classical Red recombineering method (8), which was an enormous project. If using the parallel gene editing operation platform, forty-two 96-well plates could be used simultaneously or in batches to obtain a scarless version of Keio collection.

### Conclusion

In this study, we explored the mechanism to enhance the efficiency of homologous recombination by utilizing PT-modified linear DNA sequences and designed a series of different types of donor DNAs. The results showed that PT-modified DNA could achieve highly efficient recombination through chemical transformation, with two to three orders of magnitude, allowing for chemical transformation to generate enough recombinants. Through further optimization, the highest recombination efficiency was achieved with four PT modifications and 400 bp homology arms. The recombination results of different types of repair templates indicated that the lagging strand plays an important role in recombination, and different gene locations in the genome require different design strategies. Based on this highly efficient recombination method, we developed the GIDGE method combined with CRISPR/Cas9. Using this method, various gene editing events, such as insertion, replacement, and long fragment knockout, could be completed without constructing plasmids. Based on the GIDGE method, we developed a multiple gene editing platform that enables efficient and scarless engineering of both common *E. coli* strains and wild-type strains such as *E. coli* MG1655 and *E. coli* Nissle 1917. This platform allows for parallel multi-gene editing in a 96-well plate format. Using this platform, we successfully constructed a large fragment knockout library in *E. coli* K-12, generating 65 strains with distinct genotypes. This large fragment knockout library can be used for screening purposes.

## MATERIALS AND METHODS

### Strains and cultural conditions

*E. coli* strain DH5α (ATCC 68233) served as the host strain for molecular cloning and plasmid manipulation. *E. coli* strains JM109 (ATCC 53323), MG1655 (ATCC 47076), JW128 (33), Nissle 1917 (51), and DH10B served as genetic materials in genomic editing experiments. Strains and plasmids used in this study are listed in Table S1. Luria-Bertani (LB) medium (10 g/L tryptone, 5 g/L yeast extract, and 10 g/L NaCl) was used for cell growth except where otherwise noted. The solid medium contained 20 g/L agar. Super optimal broth (SOB) medium (20 g/L tryptone, 5 g/L yeast extract, 0.5 g/L NaCl, 2.5 mM KCl, 10 mM MgCl2, and 10 mM MgSO4) was used for preparing competent cells. SOB with catabolite repression (SOC) medium (20 g/L tryptone, 5 g/L yeast extract, 0.5 g/L NaCl, 2.5 mM KCl, 10 mM MgCl2, 10 mM MgSO4, and 20 mM glucose) was used for cell recovery after heat shock or electric shock. The working concentrations of antibiotics were ampicillin (Amp) 100 µg/mL, kanamycin (Kan) 50 µg/mL, and tetracycline (Tet) 15 µg/mL. The working concentrations of inducers and inhibitors were isopropyl-β-D-thiogalactopyranoside (IPTG) 1 mM, L-arabinose 20 mM, glucose 10 g/L, and sucrose 50 g/L.

### Preparation of donor DNA

In Red recombination tests, donor DNA fragments were obtained by PCR with pUC19 plasmid as the template. The PCR products were purified with Magen HiPure Gel Pure Micro Kit. Due to their greatly different molecular weights, the template plasmid and amplified DNA could be entirely separated by agarose gel electrophoresis. When preparing donor DNA with 60 bp HAs, a pair of unmodified or PT-modified primers containing 20 bp prime sequence and 60 bp overhangs were used. The overhangs of the forward primer and the reverse primer are homologous to the upstream and downstream regions of the sequence to be replaced. The type-I donor was prepared using unmodified primers, and the type-II, type-IV, type-VI, type-VII, type-VIII, and type-X donors were prepared using a pair of PT-modified primers or a PT-modified primer and an unmodified primer. The type-III donor was prepared by digesting the type-II donor with T7 exonuclease (52, 53). The type-V donor was prepared by digesting the type-IV donor with T7 exonuclease. And the type-IX donor was prepared by digesting the type-VIII donor with T7 exonuclease. The donor DNA with 100 bp, 200 bp, 300 bp, 400 bp, and 500 bp HAs were obtained by PCR with the extracted genomic DNA of a correct recombinant as the template. In genomic editing tests, plasmid *pUC19-N20* or *pR6K19-N20* was used as the PCR template. The preparation of donor DNA for sequence deletions, sequence insertions, sequence replacements, and point mutations is illustrated in Fig. 3D through 3F.

### Red recombination tests

In Red recombination tests, plasmids *pKD46-tet* and *pUC19* were used. (i) The *pKD46-tet* plasmid was transformed into JM109 strain, generating JM109/*pKD46-tet*. (ii) The plasmid-borne strain was then made into competent cells containing overexpressed Red recombinases. (iii) 1–10 μL containing 1 µg of donor DNA amplified from the *pUC19* plasmid was transformed into JM109/*pKD46-tet*, and recombinants were selected on an LB plate containing ampicillin. (iv) Colonies growing on the plate were verified by PCR.

### Genomic editing

For genomic editing using the GIDGE method, we employed a dual-plasmid system consisting of *p15A-Red-Cas9-sgRNA* for Cas9 protein and recombinase expression, and *pR6K-N20* as a template for amplifying selection marker and pre-designed CRISPR/Cas9 target site. The editing process began with the transformation of *p15A-Red-Cas9-sgRNA* into host strains, with 1 mM IPTG added during competent cell preparation to induce Red recombinase overexpression and accumulation. Using *pR6K-N20* as template, we amplified donor DNA containing both an Amp^r^ selection marker and a 23 bp Cas9 target sequence (20 bp spacer with 3 bp PAM) designed for homologous recombination. Following purification, 1 µg donor DNA was transformed into *p15A-Red-Cas9-sgRNA*-bearing competent cells, with recombinants selected on LB plates supplemented with ampicillin, kanamycin, and glucose. PCR verification confirmed successful genomic integration in growing colonies, after which positive clones were cultured in SOC medium for 2 hours at 37°C before plating on selective media containing kanamycin, IPTG (to maintain recombinase expression), and L-arabinose (to induce Cas9). After incubation, PCR analysis of colonies validated editing outcomes, with complete workflows for sequence deletions, insertions, replacements, and point mutations detailed in Fig. S5. Correct colonies containing the *p15A-Red-Cas9-sgRNA* plasmid could be made into competent cells for a new round of genomic editing. When the last round of genomic editing was completed, the *p15A-Red-Cas9-sgRNA* plasmid was removed. In the absence of antibiotic selection pressure, plasmid-containing bacteria gradually lose plasmids due to the metabolic burden of replication and maintenance, favoring plasmid-free progeny. Concurrently, the *sacB* gene on the plasmid induces production of cytotoxic levan in sucrose-containing LB media, creating a counter-selection system that eliminates plasmid-retaining cells while allowing plasmid-cured strains to survive (54, 55). One correct colony was inoculated into an antibiotic-free LB medium and cultured for 6–8 hours at 37℃, and 2–5 μL of the culture was streaked on an LB plate containing sucrose. Generally, colonies growing on the plate were plasmid-free.

### High-throughput genomic editing

In high-throughput genomic editing, plasmids *p15A-Red-Cas9-sgRNA* and *pR6K19-N20* were used. (i) The *p15A-Red-Cas9-sgRNA* plasmid was transformed into MG1655 strain, generating *MG1655/p15A-Red-Cas9-sgRNA*. (ii) The plasmid-borne strain was then made into chemically competent cells containing overexpressed Red recombinases. Aliquot (on ice) 25 µL of the cell suspension into a sterile 96-well PCR plate. Cells are now ready for transformation or could be stored below −70℃ for transformation later. (iii) Prepare donor DNA fragments in a 96-well PCR plate by PCR with plasmid pR6K19-N20 as the template. Test the quality of donor DNA fragments by taking 5 µL of the PCR products for agarose gel electrophoresis. Unqualified donor DNA must be replaced. (iv) Pipette 2.5 µL of each donor DNA into the 96-well PCR plate containing competent cells. (v) Place the 96-well PCR plate on ice for 30 minutes. Heat shock at exactly 42℃ for exactly 1 minute. Place on ice for 2 minutes. (vi) Pipette 100 µL of SOC medium preheated at 37℃ into each well. (vii) Transfer all mixtures to a sterile 96-well culture plate. (viii) Pipette 875 µL of SOC medium preheated at 37℃ into each well. Incubate the 96-well culture plate at 37℃ for 2 hours. (ix) Add 1 µL of 100 µg/mL Amp and 1 µL of 50 µg/mL Kan into each well. Shake the cultures overnight at 37℃. (x) Pipette 100 µL of each culture into a new sterile 96-well culture plate. Add 900 µL of LB medium and 1 µL of 50 µg/mL Kan into each well. Shake the cultures at 37℃ for 2 hours. (xi) Add 10 µL of 100 mM IPTG and 20 µL of 1 M L-arabinose into each well. Shake the cultures at 37℃ for 3 hours. (xii) Pipette 50 µL of each culture into a new sterile 96-well culture plate. Add 950 µL of LB medium, 20 µL of 1 M L-arabinose, and 1 µL of 50 µg/mL Kan into each well. Shake the culture overnight at 37℃. (xiii) Take 1 µL of each culture for PCR verification. (xiv) Pipette 500 µL of 50% glycerol into each well. Now the 96-well culture plate could be stored below −70℃.

## SUPPLEMENTAL MATERIAL

Supplemental material aem.00193-25-s0001.docx Figures S1 to S9; Table S1.

Graphical abstract aem.00193-25-s0002.tif Visual schematic of the study.

ASM does not own the copyrights to Supplemental Material that may be linked to, or accessed through, an article. The authors have granted ASM a non-exclusive, world-wide license to publish the Supplemental Material files. Please contact the corresponding author directly for reuse.
