# Supplementary material for: A recombineering-based platform for high-throughput genomic editing in Escherichia coli
Source: Appl Environ Microbiol. 2025 Jun 12;91(7):e00193-25. doi: 10.1128/aem.00193-25 (PMC12285239; doi:10.1128/aem.00193-25)
Supplement: Supplemental material — Figures S1 to S9; Table S1. [file aem.00193-25-s0001.docx]

*Supplementary Material*

**A recombineering-based platform for high-throughput genomic editing in *Escherichia coli***

Zeyu Liang ^b, §^, Chaoyong Huang ^b, §^, Yitian Li ^b^, Chao Yang ^b^, Ning Wang ^b,^ ^†,^ *, Xiaoyan Ma ^a, b, c,^ *,

Yi-Xin Huo ^a, b, c^

^a^ *Center for Future Foods, Muyuan Laboratory, 110 Shangding Road, Zhengzhou 450016, Henan Province, China;*

^b^ *Key Laboratory of Molecular Medicine and Biotherapy, Aerospace Center Hospital, School of Life Science, Beijing Institute of Technology, 100081 Beijing, China;*

^c^ *Beijing Institute of Technology (Tangshan) Translational Research Center, Hebei 063611, China*

* Corresponding authors.

*E-mail addresses:* *wangning@ibcas.ac.cn (Ning Wang); xyma@bit.edu.cn (Xiaoyan Ma)*

^§^ These authors contributed equally to this work.

^†^ Present address: State Key Laboratory of Forage Breeding-by-Design and Utilization, Institute of Botany, Chinese Academy of Sciences, Beijing, 100093, China.


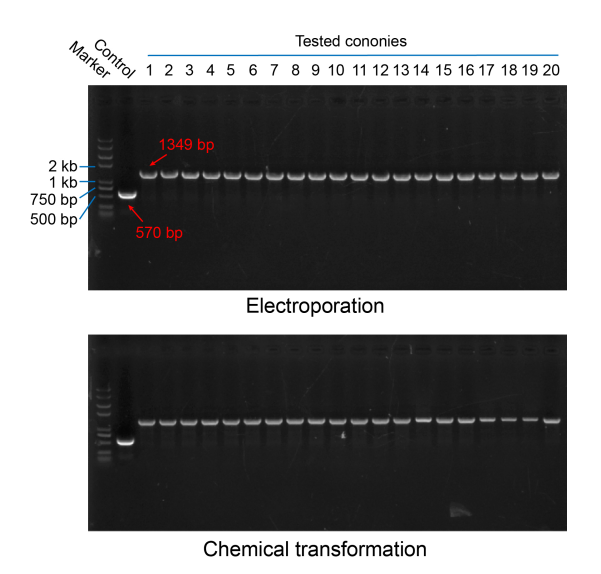


**Supplementary Fig. S1** The PCR results of Red recombination tests by electroporation and chemical transformation.


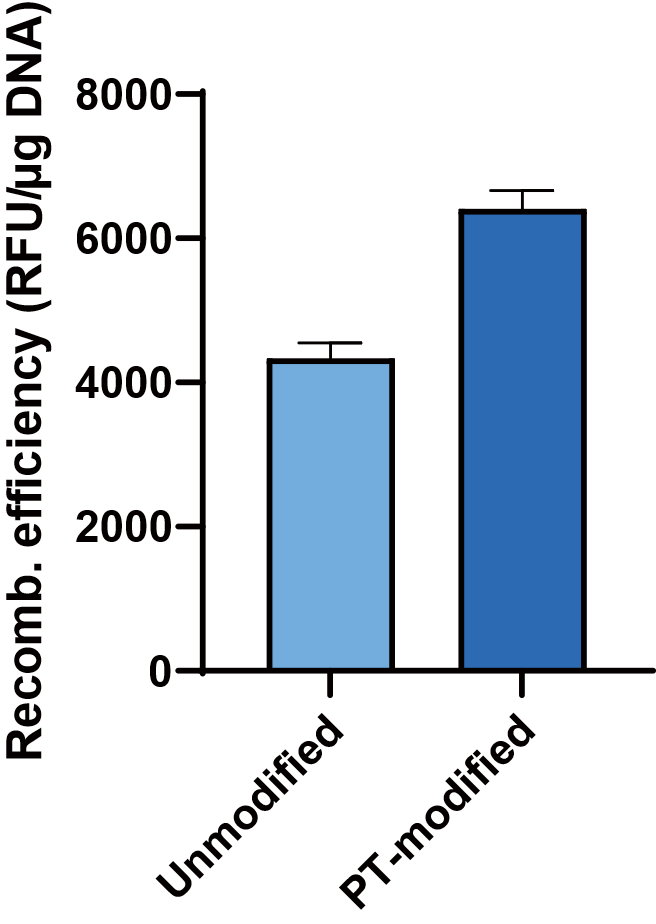


**Supplementary Fig. S2** Results of Red recombination tests using donor DNA with a 400 bp homologous arm, either unmodified or PT-modified.


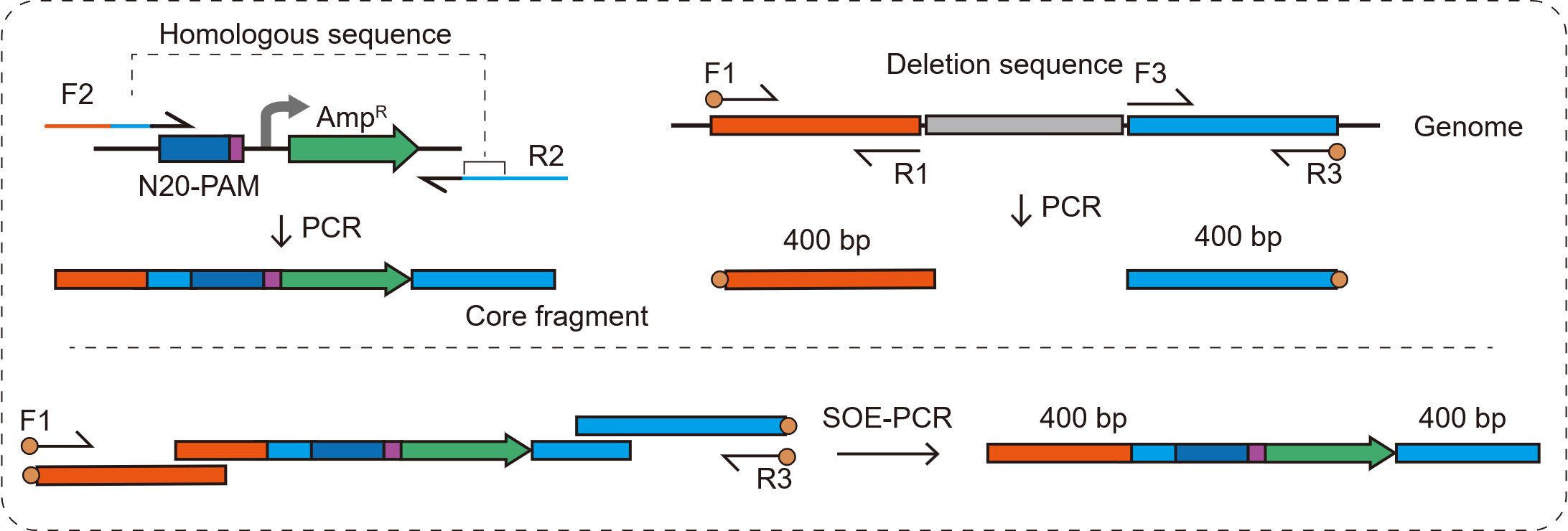


**Supplementary Fig. S3** The preparation of donor DNA with long HAs.


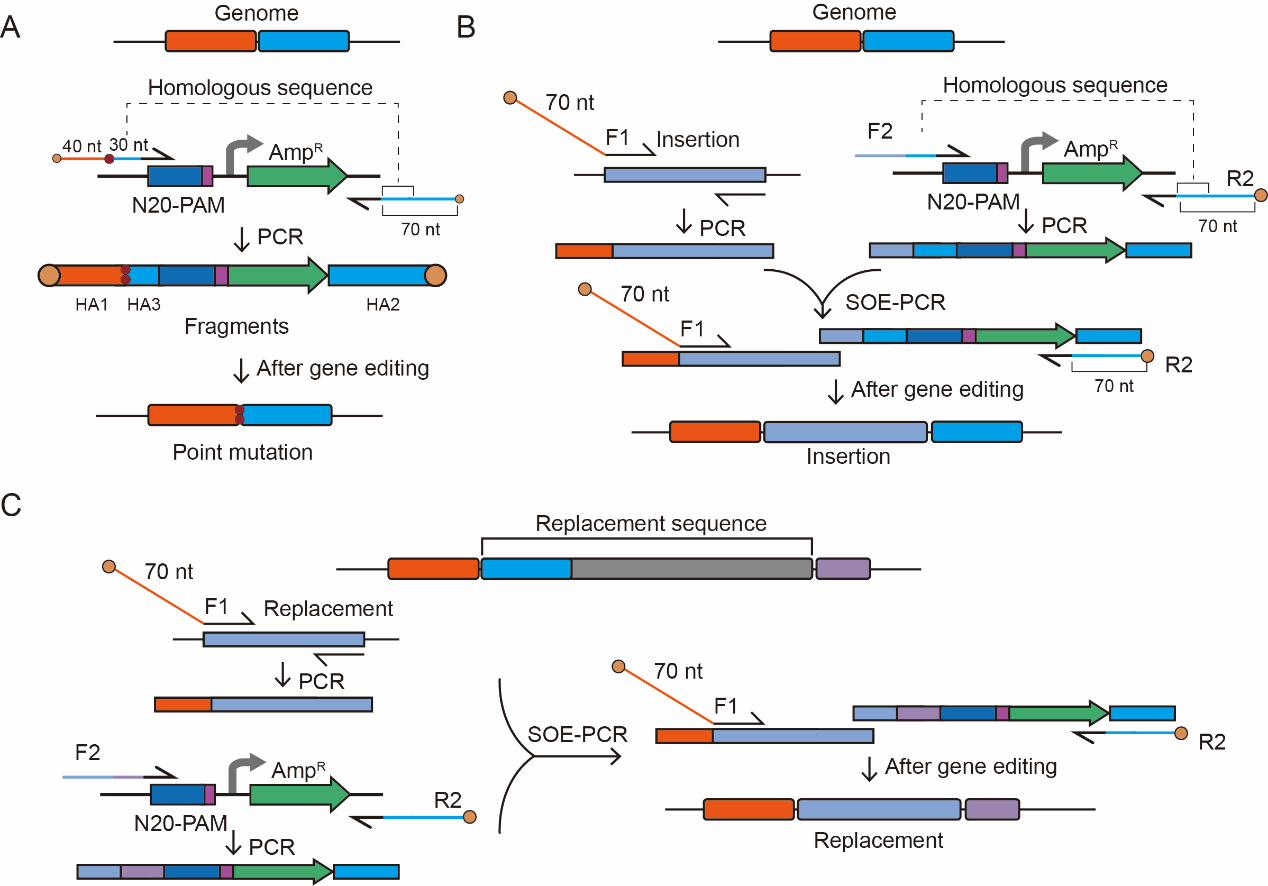


**Supplementary Fig. S4** The preparation of different donor DNA to achieve various genome editing.


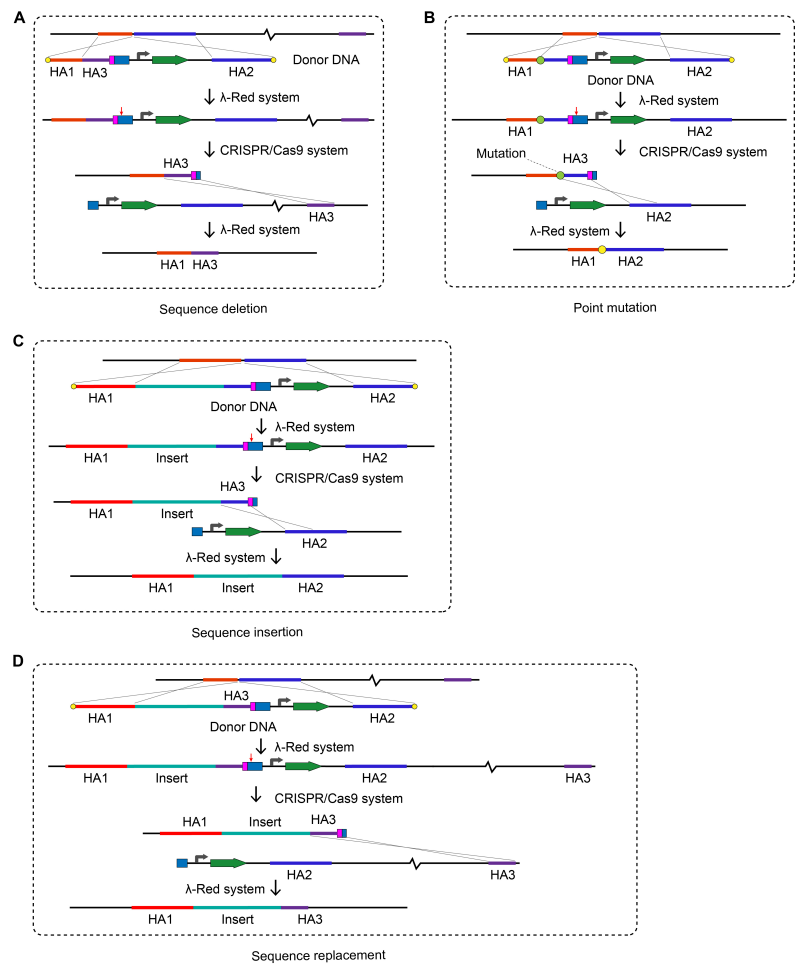


**Supplementary Fig. S5** Schematic diagram of different types of genomic editing.


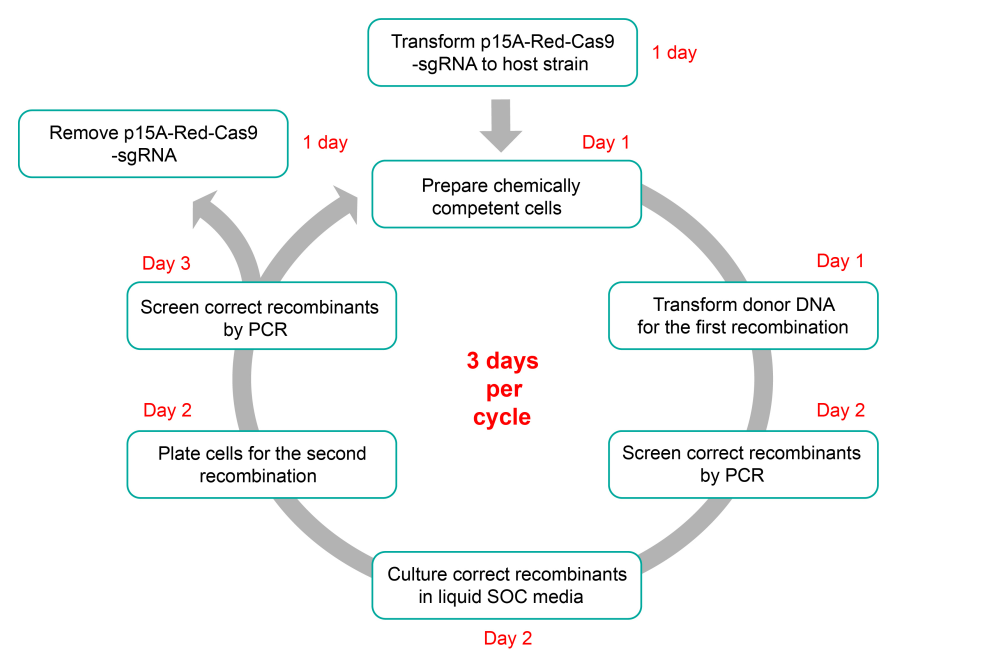
 **Supplementary Fig. S6** The simplified processes of genomic editing by the GIDGE method.


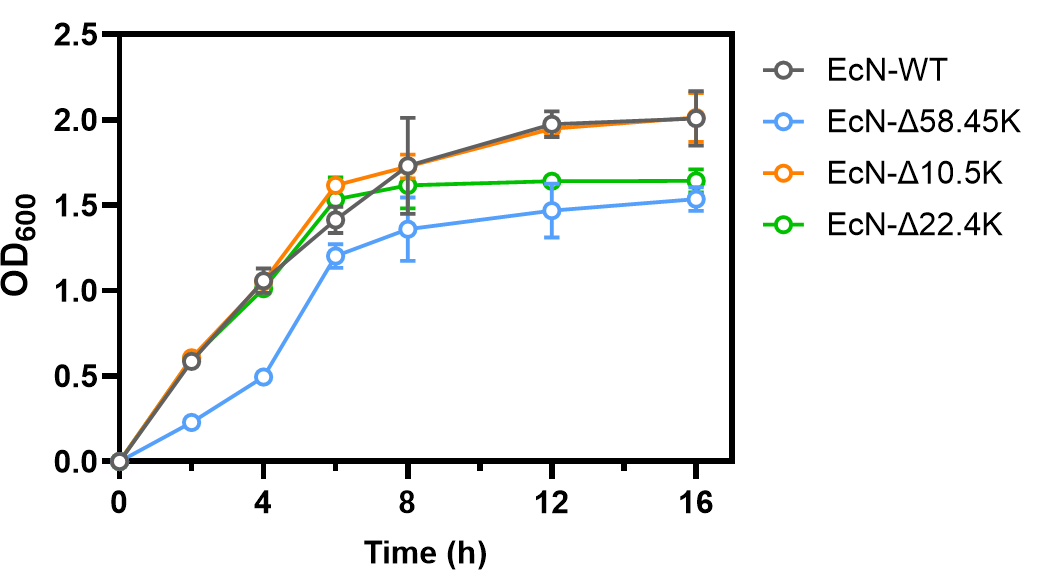


**Fig. S7.** The growth condition of the mutant strains and wild-type EcN.


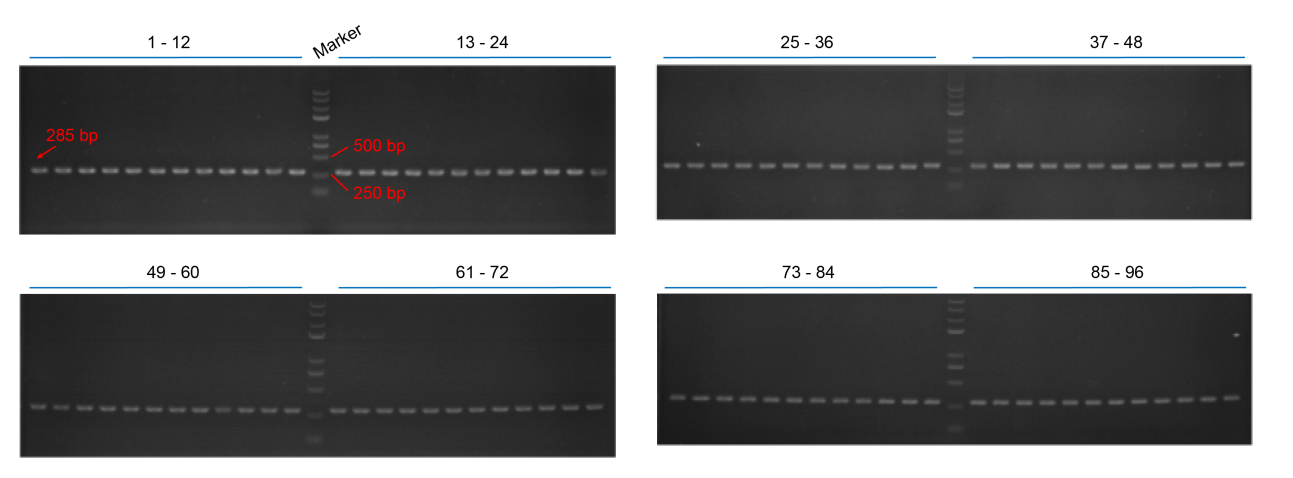


**Supplementary Fig. S8** The results of lacZ deletion test using the GIDGE-based parallel genomic operation platform.


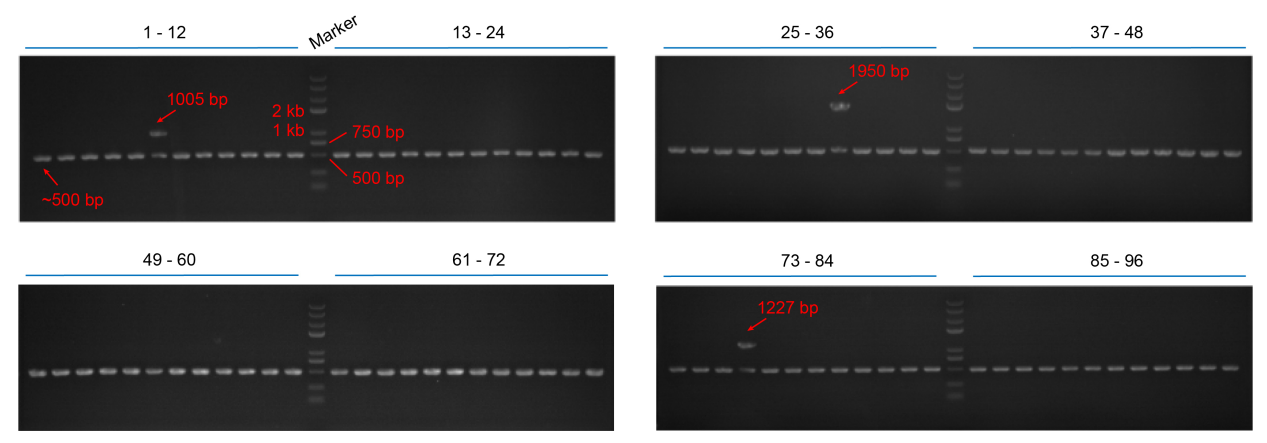


**Supplementary Fig. S9** The results of deleting 96 genes using the GIDGE-based parallel genomic operation platform.

Table S1. Strains and plasmid used in this study

| Strain and plasmid | Description | Source |
| --- | --- | --- |
| Strain |  |  |
| DH5α | *F^–^ λ^–^ endA1 glnV44 thi-1 recA1 relA1 gyrA96 deoR nupG purB20 φ80dlacZΔM15 Δ(lacZYA-argF) U169 hsdR17 (r_K_ ^–^m_K_ ^+^)* | Our lab. |
| JM109 | *endA1 glnV44 thi-1 recA1 relA1 gyrA96 Δ(lac-proAB) e14^–^ [F' traD36 proAB^+^ lacI^q^ lacZΔM15] hsdR17*(r_K_ ^–^m_K_ ^+^) | Our lab. |
| MG1655 | *F^–^ λ^–^ ilvG^–^ rfb-50 rph-1* | Our lab. |
| Nissle 1917 | *O6: K5: H1 EndA^+^ Dcm^+^* | Our lab. |
| JW128 | *MG1655 (ΔaraBAD::Tet^r^ ΔmcrCB-hsdSMR-mrr mcrA^–^ endA^–^ recA^–^ )* | Our lab. |
| DH10B | *F^-^ mcrA Δ(mrr-hsdRMS-mcrBC) φ80lacZΔM15 ΔlacX74 nupG recA1 araD139 Δ(ara-leu)7697 galE15 galK16 rpsL(Str r) endA1 λ^-^* | Our lab. |
| Plasmids |  |  |
| pKD46-tet | Used to express lambda Red recombinases | This study |
| pUC19 | Used as the template to amplify donor DNA for recombination tests | Our lab. |
| p15A-Red-Cas9-sgRNA | Used for genomic editing | This study |
| pUC19-N20 | Used as the template to amplify donor DNA for genomic editing | This study |
| pR6K19-N20 | Used as the template to amplify donor DNA for genomic editing | This study |
