# Supplementary figures and images for: A recombineering-based platform for high-throughput genomic editing in Escherichia coli
Source: Appl Environ Microbiol. 2025 Jun 12;91(7):e00193-25. doi: 10.1128/aem.00193-25 (PMC12285239; doi:10.1128/aem.00193-25)

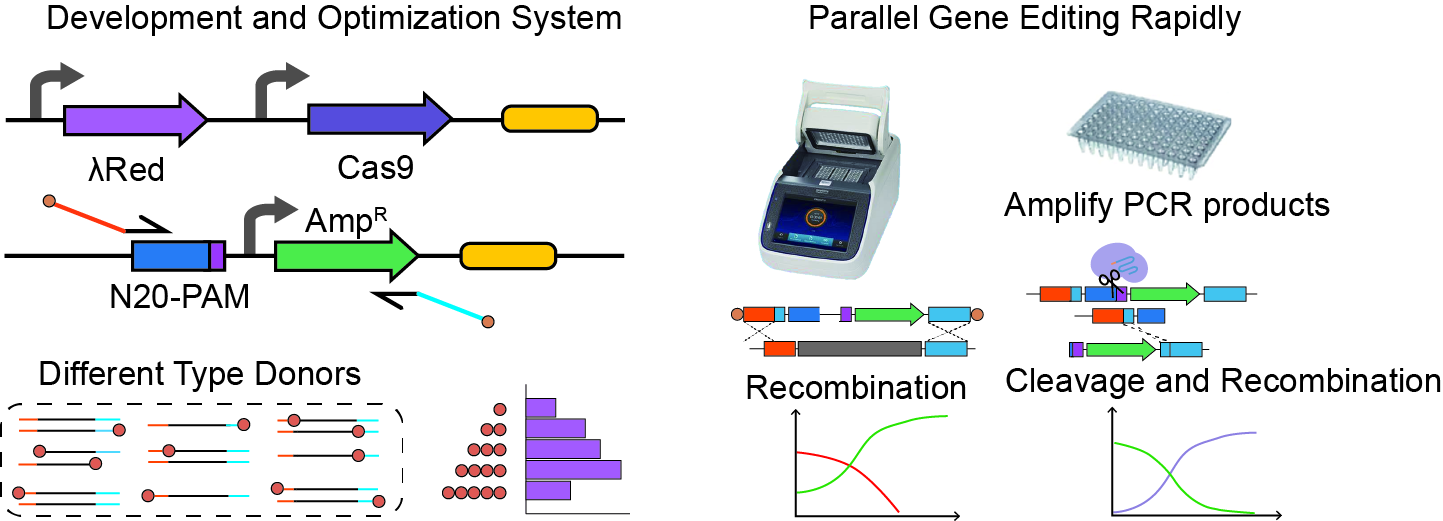

Supplement: Graphical abstract — Visual schematic of the study. [file aem.00193-25-s0002.tif]
